# Tracheal Replacement: A Scoping Review

**DOI:** 10.1055/s-0044-1792126

**Published:** 2024-11-12

**Authors:** Darin T. Johnston, David B. Powers, Matthew G. Hartwig, Russel R. Kahmke, Linda C. Cendales

**Affiliations:** 1 Uniformed Services University, Craniomaxillofacial Trauma & Reconstruction, Oral & Maxillofacial Surgery, David Grant Medical Center, Travis AFB, California; 2 Division of Plastic, Maxillofacial and Oral Surgery, Department of Surgery, Duke University Hospital, Durham, North Carolina; 3 Division of Cardiovascular and Thoracic Surgery, Department of Surgery, Duke University Hospital, Durham, North Carolina; 4 Department of Head and Neck Surgery and Communication Sciences, Duke University Hospital, Durham, North Carolina

**Keywords:** microvascular, reconstruction, transplant, surgery, airway stenosis, trachea

## Abstract

**Objective** To summarize patient characteristics and outcomes for the historical and current methods of long-segment tracheal replacement in humans.

**Materials and Methods** A single reviewer screened the abstracts and full texts using Covidence for file management. Studies published in English that reported human subjects with circumferential or near-circumferential (>270 degrees) cervical tracheal replacements were included. Articles with subjects treated with primary anastomosis alone, retracted articles, abstracts, expert opinion articles, and conference presentations were excluded.

**Results** A total of 32 articles were included in the review reporting 156 cases of long-segment tracheal replacement including synthetic (alive at 1–8 years *n* = 6/64), regenerative medicine (dead at 15 days–55 months *n* = 4, not reported *n* = 6), cadaveric tracheal allograft (alive at 5 months–10 years *n* = 32/38), aortic allograft (alive at 6–85 months *n* = 12/16), free tissue transfer (alive at 6–108 months *n* = 13/21), allotransplantation (alive at 6–24 months *n* = 5/8), and vascular composite allograft (VCA) (alive at 20 months *n* = 1/1).

**Conclusion** Silicone and Marlex prostheses have poor long-term outcomes. The cadaveric tracheal allograft can only replace near-circumferential tracheal defects and is therefore limited to benign tracheal pathology. Inadequate structural support plagues the aortic allograft and often requires numerous invasive procedures and maintenance of an intraluminal stent. A lack of mucociliary clearance exists in all methods of tracheal replacement except cadaveric tracheal allograft and VCA and can cause fatal mucous plugging and chronic pulmonary infections. VCA and allotransplantation require long-term immunomodulation therapy.

Tracheal resection is performed for patients of all ages due to tracheal stenosis and other benign tracheal pathologies, tracheoesophageal fistulae (TEFs), tracheal neoplasms, and infiltrating lesions such as thyroid malignancy. The defect can be circumferential, near circumferential with the preserved posterior tracheal wall, or only involve a portion of the cartilaginous structure. In circumferential and near-circumferential resections, the reconstructive options are numerous. Generally, tracheal defects less than 6 cm or 50% of tracheal length in adults and 33% in children can be reconstructed with primary anastomosis following mobilization of the cervical and mediastinal trachea. Before 1968, the cutoff for primary anastomosis in adults was 3 cm.

Modern reconstruction of circumferential or near-circumferential (>270 degrees) defects not amenable to primary anastomosis has been performed for more than 50 years using various tracheal replacement methods and materials. The first experimental materials included stainless steel mesh, polyethylene, Lucite cylinder, acrylic tube, glass tube, silicone tube, Ivalon sponge, and Marlex mesh. 1 Complications related to the prosthesis included innominate artery erosion, sepsis, suture line dehiscence, stenosis, and obstruction. 1 2 3 4

Biologic solutions include aortic autograft aortic allograft, cadaveric tracheal homografts, free tissue transfer, staged allotransplantation, and vascular composite allograft (VCA). 5 6 7 8 9 10 11 12 13 14 15 16 17 18 19 20 21 Tissue-engineered tracheal replacements have been employed on both synthetic and cadaveric tracheal frameworks. 22 23 24 25

Goals of tracheal replacement are to re-establish a structure that maintains airway patency under dynamic pressure gradients, remains longitudinally flexible, integrates into the adjacent tissues, and provides physiologic mucus management with functional respiratory cilia. 21 Additional factors influencing clinical practice and research of any modality for tracheal replacement relate to a patient's quality of life, immunomodulation, monitoring, the indication for additional invasive interventions, duration of airway stents, and long-term tracheostomies.

The purpose of this scoping review is to summarize patient characteristics and outcomes for the historical and current methods of long-segment tracheal replacement in humans.

## Materials and Methods

### Literature Search Strategy

A search was performed on March 18, 2022, using MeSH terms in PubMed “Tracheal Transplantation,” “Tracheal Replacement,” “Tracheal Substitute,” “Tracheal Regeneration,” and “Tracheal tissue engineering.” Filters were applied to exclude results not in the English language and without available abstracts. Covidence software was used for file management.

### Selection Criteria

Studies published that reported human subjects with circumferential or near-circumferential (>270 degrees) cervical tracheal replacements were included. Reviews with additional patient information that met the selection criteria were also included. Articles with subjects treated with primary anastomosis alone, retracted articles, abstracts, expert opinion articles, and conference presentations were excluded.

### Data Extraction and Analysis

Extracted data points included the year of the surgery, country where it was performed, sex and age of the patient, resected tracheal pathology, resection type (circumferential or near-circumferential) and length, reconstruction method, cause of death, and duration of follow-up. As a scoping review, data points were aggregated, but analysis was not performed.

## Results

### Clinical Studies

**Fig. 1:**
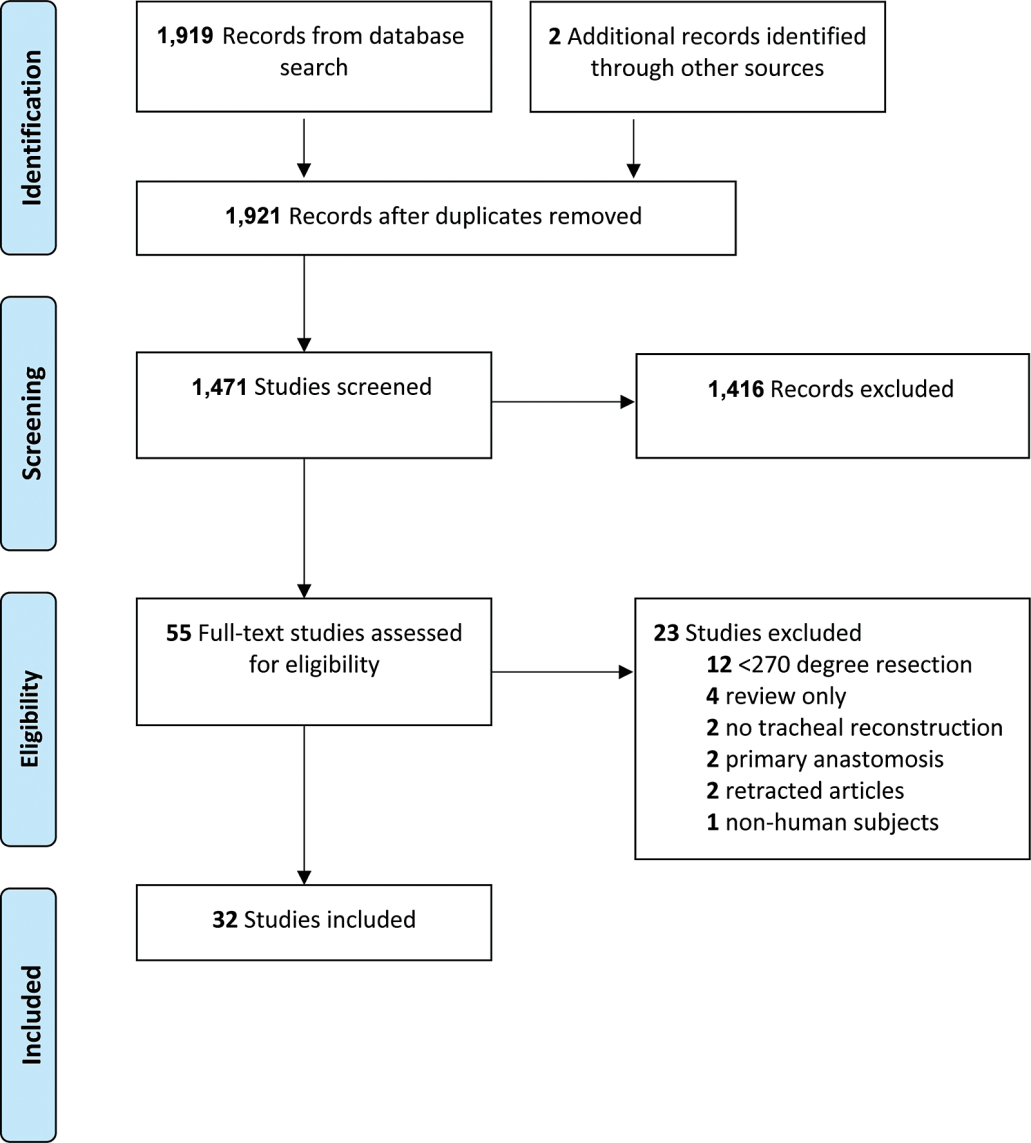
Flow diagram for study selection (as adapted from the PRISMA statement).

There were 1,938 references identified. Thirty articles were included (PRISMA diagram, Fig. 1 ); 156 cases were reported from Argentina, Australia, Belgium, Canada, China, France, Germany, Italy, Japan, Poland, Portugal, South Africa, Sweden, Thailand, United Kingdom, and United States. Single case reports comprised 11 included articles and the remaining 19 were case series. The earliest included tracheal replacement was in 1963 and the most recent was in 2020. Table 1 lists the key characteristics of all included studies. Collected data points for each type of tracheal reconstruction, summarized in Table 2 , included total patients meeting inclusion criteria, years of publications, countries where treatment was rendered, pathology necessitating tracheal resection, resection type, length of resection, and mortality outcomes. 1 2 3 4 5 6 7 8 9 10 11 12 13 14 15 16 17 18 19 20 21 22 23 24 26 27 28 29 30 31 32

**Table 1:** Key characteristics of all included studies

Author	Reported	Performed	Country	Design	No. of patients	Age (y)	Pathology	Resection	Length	Reconstruction type	Follow-up	Reported cause of death
Olmedo et al 2	1982	1971–1979	Argentina	Case report	1	16	ACCa	C	8 cm	Silicone prosthesis	7 d	Airway dehiscence
Toomes et al 3	1985	1979–1985	Germany	Case series	5	49–58	SCCa (2), ACCa (1), stenosis (1), tracheomalacia (1)	C	6–8 cm	Silicone prosthesis	15 d–10 mo	Cancer (2), hemorrhage (2), sepsis (1)
Neville et al 1	1990	1970–1988	United States	Case series	27	NR	Stenosis (20), malignant tracheal tumor (7)	C	NR	Silicone prosthesis	1–8 y	Alive (6), cancer (2),unrelated (3), NR (16)
Neville et al 1	1990	1970–1988	United States	Case series	21	NR	Stenosis (15), malignant tracheal tumor (6)	Near-C	NR	Silicone prosthesis	6–24 mo	Unrelated (6), cancer (6), NR (9)
Maziak et al 4	1996	1963–1995	Canada	Case series	5	NR	ACCa	C	NR	Marlex prosthesis	<1 mo	Hemorrhage (2), NR (3)
Jacobs et al 11	1996	NR	UK	Case series	24	<1–18	Stenosis	Near-C	NR	Cadaveric tracheal allograft	5–120 mo, NR (4)	Alive (20), unknown (4)
Beldholm et al 18	2003	NR	Australia	Case report	1	43	ACCa	C	6 cm	Free tissue transfer	16 mo	Cancer
Olias et al 17	2005	2003	Portugal	Case report	1	25	Stenosis	C	4.5 cm	Free tissue transfer	6 mo	Alive
Azorin et al 5	2006	2004	France	Case report	1	68	SCCa	C	8 cm	Aortic autograft	6 mo	Pulmonary infection
Yu et al 13	2006	NR	United States	Case report	1	63	Recurrent thyroid carcinoma	C	6.5 cm	Free tissue transfer	6 mo	Alive
Kunachak et al 32	2007	NR	Thailand	Case series	4	2–40	Stenosis	Near-C	1–6.5 cm	Cadaveric tracheal allograft	18–20 mo, NR (1)	Alive (3), unknown(1)
Davidson et al 6	2009	2007	South Africa	Case report	1	33	TEF	C	6 cm	Aortic autograft	10 d	Airway dehiscence
Maciejewski et al 14	2009	NR	Poland	Case report	1	24	Recurrent thyroid carcinoma	C	7 cm	Free tissue transfer	24 mo	Alive
Delaere et al 19	2010	2008	Belgium	Case report	1	55	Stenosis	C	4.5 cm	Allotransplantation	1 y	Alive
Wurtz et al 9	2010	2005–2007	France	Case series	6	17–52	ACCa (5), MECa (1)	C	8.5–11 cm	Aortic allograft	26–45 mo	Alive (4), cancer (1), hemorrhage (1)
Kanemaru et al 25	2010	NR	Japan	Case series	3	39–71	Stenosis	C	NR	In situ synthetic trachea	6 mo	Alive
Propst et al 10	2011	2001–2009	United States	Case series	10	2–16	Stenosis	Near-C	2–8 cm	Cadaveric tracheal allograft	<1–90 mo	Alive (9),airway dehiscence (1)
Delaere et al 33	2012	2008–2011	Belgium	Case report	1	26	Stenosis	C	8 cm	Allotransplantation	6 mo	NR
Xu et al 20	2014	2011–2013	China	Case report	1	51	Malignant tracheal tumor	C	7 cm	Allotransplantation	6 mo	Alive
Fabre et al 28	2015	2006–2015	France	Case series	15	23–68	ACCa (9), SCCa (3), thyroid cancer (1), TEF (1), tracheomalacia (1)	C	8–12 cm	Free tissue transfer	<1–108 mo	Alive (9), ARDS (4), cancer (1), hemorrhage (1)
Zhang and Liu 29	2015	NR	China	Case report	1	31	ACCa	C	5 cm	Metal stent	NR	NR
Hamilton et al 30	2015	2010	UK	Case report	1	10	Stenosis	C	7 cm	In situ cadaveric scaffold	5 y	Alive
Martinod et al 27	2017	2010–2011	France	Case series	2	33–58	Stenosis (2)	Near-C	5 cm	Aortic allograft	55–67 mo	Alive
Elliot et al 23	2017	NR	UK	Case report	1	15	Tracheomalacia	C	NR	Ex vivo cadaveric scaffold	15 d	Respiratory arrest
Martinod et al 7	2018	2010–2017	France	Case series	5	24–64	Stenosis (3), anaplastic thyroid carcinoma (1), papillary thyroid carcinoma (1)	C	NR	Aortic allograft	9–85 mo	Alive
Thomet et al 16	2018	NR	Switzerland	Case series	2	35–70	ACCa (1), chondrosarcoma (1)	C	6 cm	Free tissue transfer	27–36 mo	Alive (1), cancer (1)
Fux et al 22	2020	2011–2012	Sweden	Case series	3	37	MECa (1), ACCa (1), TEF (1)	C	NR	Ex vivo synthetic scaffold	3.5–55 mo	Hemorrhage (1), obstruction (1), unknown (1)
Menna et al 8	2021	2021	Italy	Case report	1	50	Stenosis	Near-C	NR	Aortic allograft	2 mo	Alive
Genden et al 35	2022	2020	United States	Case report	1	56	Stenosis	C	8 cm	Vascular composite allograft	20 mo	Alive

Abbreviations: ACCa, adenoid cystic carcinoma; ARDS, acute respiratory distress syndrome; C, circumferential; MECa, mucoepidermoid carcinoma; NR, not reported; SCCa, squamous cell carcinoma; TEF, tracheoesophageal fistula.

**Table 2:** Summary of included studies

	Total patients	Publication year range	Countries	Pathology treated	Resection type	Length range	Mortality
Synthetic	60	1982–2015	Argentina, Canada, China, Germany, Japan, United States	Stenosis (35), malacia (2), cancer (27)	Circumferential (39), near-circumferential (22)	5–8 cm (NR 54)	Alive at 1–8 y (6); dead at 7 d–10 mo, airway related (6); dead 2–18 mo, not airway related (19); NR (29)
Regenerative medicine							
In situ synthetic scaffold, ex vivo synthetic scaffold, in situ cadaveric scaffold, ex vivo cadaveric scaffold	8	2010–2020	Japan, Sweden, UK	Stenosis (4), malacia (1), TPF (1), cancer (2)	Circumferential	7 cm (NR 6)	Dead at 15 d–3 mo, not airway related (2); dead at 32–55 mo, airway related (2)
Cadaveric tracheal allograft	38	1996–2011	Thailand, UK, United States	Stenosis	Near-circumferential	1–8 cm (NR 24)	Alive at 5–120 mo (32); dead at 1 mo, airway related (1); cause of death and timing, NR (4); NR (1)
Aortic allograft	16	2006–2021	France, Italy, South Africa	Stenosis (6), TEF (1), cancer (9)	Circumferential (13), near-circumferential (3)	5–11 cm (NR 6)	Alive at 6–85 mo (12); dead at 45 mo, cancer (1); dead at 6 mo, pulmonary disease (1); dead at 10–26 mo, dehiscence, hemorrhage (2)
Free tissue transfer	21	2003–2018	Australia, France, Poland, Portugal, United States	Stenosis (1), TEF (1), malacia (1), cancer (18)	Circumferential	4.5–12 cm	Alive at 6–108 mo (13); dead at 1–45 mo, pulmonary disease (4); dead at 16–27 mo, cancer (3); dead at 6 mo, hemorrhage (1)
Allotransplantation							
Forearm SR greater omentum SR	8	2010–2014	Belgium, China	Stenosis (4), cancer (4)	Circumferential	4.5–9 cm	Alive at 6–24 mo (5); other (1); NR (2)
Vascular composite allograft	1	2021	United States	Stenosis	Circumferential	8 cm	Alive at 20 mo

Abbreviations: NR, not reported; SR, secondary revascularization; TEF, tracheoesophageal fistula; TPF, tracheopulmonary fistula.

### Synthetic Prosthesis

A Marlex prosthesis was placed in five patients between the years 1963 and 1995. The age, sex, and tracheal defect lengths were not reported. All were placed following circumferential resection for adenoid cystic carcinoma (ACCa). Two patients died from airway hemorrhage within 1 month of tracheal replacement. Outcomes for the remaining three patients were not reported. 4

A silicone prosthesis was placed in 54 patients with findings reported between the years 1970 and 1988. There were four male patients and two female patients ranging in age from 16 to 58 years. Sex and age were not reported in 48 cases. Circumferential tracheal resections were performed for malignant airway tumors ( *n* = 11), stenosis ( *n* = 21), and malacia ( *n* = 1). Near-circumferential resections were performed for malignant airway tumors ( *n* = 6) and stenosis ( *n* = 15). Four patients experienced dehiscence, airway hemorrhage, or sepsis related to the prosthesis between 1 week and 10 months following reconstruction. Nineteen patients died from airway cancer or other causes not related to prosthesis between 2 and 24 months. Six patients were still alive at 1 to 8 years. Outcomes were not reported for 25 patients. 1 2 3

The most recent synthetic tracheal replacement reported was performed for a 31-year-old man who underwent a circumferential resection 5 cm in length for ACCa. A metal stent was wrapped with a pedicled pulmonary tissue flap. No outcomes were reported. 29

### Regenerative Medicine

Tracheal replacement using in situ tissue engineering with a synthetic scaffold was reported in 2010 for one male patient and two female patients with tracheal stenosis ranging in age from 39 to 71 years. A Marlex mesh with spiral stent was covered with a porcine collagen sponge and processed to create an artificial trachea. Circumferential tracheal resections and placement of a T-tube were performed during the first stage. During the second stage, the artificial trachea was coated with autogenous venous blood and basic fibroblast growth factor. The artificial trachea was then implanted. All three patients were alive at 6 months without immunomodulation. Tissue biopsies to confirm ciliated respiratory epithelium were not performed. 31

Tracheal replacement using ex vivo tissue engineering with a synthetic scaffold was performed between 2011 and 2012. Tracheal resections were performed for a 37-year-old man and 30-year-old man with malignant airway tumors, and a 22-year-old woman with an iatrogenic tracheopleural fistula. A synthetic tracheal scaffold made of nanocomposite polymers was seeded with autogenous bone marrow-mononuclear cells (BM-MNCs) and processed in a bioreactor. Immediately prior to implantation, the engineered trachea was again seeded with BM-MNCs, human transforming growth factor β (TGF-B), granulocyte colony-stimulating factor (GCSF), and synthetic erythropoietin. Biopsies of the engineered trachea at the time of implantation did not contain cells. The grafts did not integrate with the surrounding tissues. Each patient underwent interventions to manage tracheal fistulas, airway collapse, obstructive granulation tissues, graft dehiscence and migration, mediastinitis, and thromboembolic events. The patients died from airway-related causes 3.5 to 55 months following tracheal replacement. 22

Tracheal replacement using in situ tissue engineering with a cadaveric scaffold was performed in 2010 for a 12-year-old boy with congenital tracheal stenosis. During tracheal resection, a decellularized donor cadaveric trachea acquired from a tissue bank was coated with BM-MNCs, human recombinant erythropoietin, GCSF, and TGF-B. Respiratory epithelium stamp grafts were harvested from the resected trachea and placed as free grafts in the donor tracheal lumen. An absorbable intraluminal stent was secured and the construct was implanted and wrapped in omentum. Multiple stent replacements were required postoperatively including a final nitinol stent at 5 months. Ciliated respiratory epithelium with normal beat pattern was identified 15 months following implantation. The patient was alive after 4 years and had returned to school. 24 30

Tracheal replacement using ex vivo tissue engineering with a cadaveric scaffold performed on a 15-year-old girl with congenital tracheal stenosis was reported in 2010. A decellularized cadaveric trachea acquired from a tissue bank was processed in a bioreactor with autologous stem cells and autologous respiratory epithelium cells. The engineered trachea was implanted without a stent following circumferential tracheal resection. The tracheostomy tube proximal to the graft was maintained. The postoperative period was unremarkable. However, 15 days following implantation ventilatory compromise due to a narrowed graft lumen led to prolonged respiratory arrest and cerebral edema. She died when ventilatory support was discontinued. 23

### Cadaveric Tracheal Allograft

Cadaveric tracheal allografts from a tissue bank decellularized in formalin were used in 34 patients, and 4 patients received a cryopreserved cadaveric tracheal homograft. There were 4 male patients, 6 female patients, and 28 patients with unreported sex. The age range for 37 patients was younger than 1 year to 18 years. One patient was 40 years old. The first reported case was in 1996. The most recent reported case was in 2007. All 38 resections were near circumferential and were performed for tracheal stenosis. Resection length was 3 to 6.5 cm in 14 patients and not described for 30 patients. The reported outcomes included graft infections ( *n* = 10), removal of stent within the first postoperative year ( *n* = 10), decannulation ( *n* = 6), and ciliated respiratory epithelium demonstrated on biopsy. one patient died within the first month due to graft dehiscence. The timing and cause of death were not reported for four patients. No outcomes were reported for one patient. The remaining 32 patients were still living at 6 months to 10 years of follow-up. 10 11 12 32

### Aortic Allograft and Autograft

Aortic allografts were placed in 14 patients and aortic autografts were placed in 2 patients. There were 11 male patients and 5 female patients ranging in age from 17 to 68 years. The first case was an autograft reported in 2004. The most recent report was in 2017. Circumferential tracheal resections were performed for malignant airway tumors ( *n* = 9), stenosis ( *n* = 3), and acquired TEF ( *n* = 1). Near-circumferential resections were performed for stenosis ( *n* = 3). Reported tracheal defect lengths ranged from 5 to 11 cm with defect length not reported for six patients. The presence of ciliated respiratory epithelium was not confirmed. Deaths related to airway reconstruction (dehiscence, hemorrhage, infection) occurred in three patients between 10 days and 26 months, and one patient died from recurrent airway cancer 45 months after tracheal reconstruction. There were 3 patients still living at 24 months and 10 patients at 2 to 7 years. Of these 13 living patients, an intraluminal stent was still required. 5 6 7 8 9 27

### Free Tissue Transfer

Tracheal reconstruction with fasciocutaneous forearm free tissue transfer requires structural support to maintain a patent airway. This single-stage procedure was performed on 11 females and 10 males, ranging in age from 23 to 70 years. The first reported case was in 2003 and the most recent in 2018. Structural support was provided by autogenous rib strips ( *n* = 18), resorbable mesh ( *n* = 2), or metal stent ( *n* = 1). Circumferential tracheal resections were performed for malignant airway tumors ( *n* = 18), acquired TEF ( *n* = 1), tracheomalacia ( *n* = 1), and stenosis ( *n* = 1). Tracheal defect lengths ranged from 4.5 to 12 cm. The reported outcomes included four deaths from acute respiratory distress syndrome between 1 and 45 months, three deaths from airway cancer between 16 and 27 months, and one death from airway hemorrhage at 6 months. There were 2 patients still living at 6 months, and 11 were still living at 1 to 9 years. Airway biopsies from 10 patients demonstrated absent ciliated respiratory epithelium. 13 14 15 16 17 18 28

### Allotransplantation

In two-stage radial forearm allotransplantation, a donor trachea is harvested and wrapped in the recipient's forearm fascia for heterotopic revascularization. During the revascularization period, the patient is immunomodulated with tacrolimus, azathioprine, and methylprednisolone. Following revascularization of the donor tracheal mucosa, tracheal resection is performed and the donor trachea is transferred with the radial artery and vein concomitants to an orthotopic position to reconstruct the trachea with microvascular anastomosis. An intraluminal stent is not placed. The heterotopic revascularization was performed on three male and two female patients, ranging in age from 17 to 64 years. The procedures were performed from 2008 to 2011. In one patient, the immunomodulation regimen was withdrawn prematurely causing loss of the donor trachea in the heterotopic position. Indications for tracheal resection in the remaining four patients were tracheal stenosis ( *n* = 3) and airway malignancy ( *n* = 1) with defects 4.5 to 9 cm in length. The reported outcomes included no patient deaths during 6 to 24 months of follow-up. The presence of ciliated respiratory epithelium was not confirmed. Withdrawal of immunomodulation in the orthotopic position resulted in necrosis of donor tracheal mucosa and cicatricial narrowing of the lumen necessitating tracheostomy in one patient. 19 26 33

In two-stage greater omentum allotransplantation, a donor trachea is harvested and wrapped in the recipient's greater omentum for heterotopic revascularization. The tracheal lumen was secured to the abdominal skin and exposed to air for inspection and clearance of secretions. During the revascularization period, the patient is immunomodulated with tacrolimus, mycophenolate, and methylprednisolone. Following revascularization of the donor tracheal mucosa, tracheal resection is performed and the donor trachea is transferred to the orthotopic position. An intraluminal stent is not placed. Immunomodulation is continued indefinitely. The two-stage procedure was performed on three male patients, ranging in age from 50 to 62 years. The procedures were performed from 2011 to 2013. Indications for tracheal resection were airway malignancy, and the tracheal defects were 6 to 7 cm in length. The reported outcomes included no patient deaths during the first 6 months of follow-up. The presence of ciliated respiratory epithelium immediately prior to orthotopic transfer was confirmed with histopathology. 20

### Vascular Composite Allograft

In long-segment tracheal VCA, the trachea and anterior esophageal wall are harvested from a living donor and transplanted to reconstruct the trachea with microvascular anastomoses. The procedure was performed on a 56-year-old woman in 2020 for acquired tracheal stenosis. The resected trachea was 9 cm. An intraluminal stent was not placed. The reported outcomes included functioning ciliated respiratory epithelium confirmed with biopsy, no detectable free cell DNA, and chimeric repopulation of the lumen mucosa. The patient remains on immunomodulation based on tacrolimus, mycophenolate, and methylprednisolone. At 20 months of follow-up, she continues to work and live a normal life. 21 34 35

## Discussion

Reconstructing circumferential and near-circumferential tracheal defects is challenging. Many materials and techniques have been used for the past 50 years. An updated and detailed review of five methods of tracheal replacement—synthetic prosthesis, aortic and tracheal allograft, tracheal allotransplantation, tissue engineering, and composite tissue allograft—was recently provided by Etienne et al. 36

Several years following the above comprehensive review, the first long-segment VCA was reported. 21 The segmental vasculature of the tracheoesophageal complex was previously considered insurmountably complex. However, it was eventually demonstrated that preserving a portion of the donor esophagus maintained perfusion of the trachea from the cricoid to the carina. 37 38 During the first months following transplantation, a functional mucociliary elevator developed. Serial biopsies demonstrated that 75% of the respiratory mucosa was derived from the recipient. 21 This remarkable accomplishment builds on the science and ethical momentum generated by other successful VCA-type transplants such as hand, face, upper and lower limb, abdominal wall, chest wall, spine, glands, uterus, and phallus transplants. 39

Long-term immunomodulation is required by all VCA, including tracheal allotransplantation, and represents a tradeoff to these modalities. The tracheal allograft can only replace near-circumferential tracheal defects and is therefore limited to benign tracheal pathology. Inadequate structural support plagues the aortic allograft and often requires numerous invasive procedures and maintenance of an intraluminal stent. A lack of mucociliary clearance exists in all methods of tracheal replacement except VCA and can cause lethal mucous plugging and chronic pulmonary infections.

## Conclusion

Trachea reconstruction continues to evolve with important advances as investigators advance this field from nonvascularized to vascularized options. Of the reported methods, the vascularized composite allograft maintains airway patency under dynamic pressure gradients without stents, remains longitudinally flexible, integrates into the adjacent tissues, and provides physiologic mucus management with functional respiratory cilia. Notwithstanding, and like all other VCA types, the unanswered questions outnumber the answered questions.
